# Maximum carotid artery wall thickness and risk factors in a young primary prevention population

**DOI:** 10.1002/brb3.82

**Published:** 2012-08-08

**Authors:** A S Callahan, Michael Szarek, John W Patton, Anne-Sophie Sillesen, Abrill Jones, Keith Churchwell, H Douglas Holliday

**Affiliations:** 1 Meharry Medical CollegeNashville, Tennessee; 2 Vanderbilt University School of MedicineNashville, Tennessee; 3 New York UniversityNew York, New York; 4 University of CopenhagenCopenhagen, Denmark; 5 Saint Thomas HospitalNashville, Tennessee

**Keywords:** Atherogenesis, carotid wall thickness, IMT, stroke

## Abstract

Maximum carotid artery wall thickness was utilized in a primary prevention population and compared with baseline risk factors. Carotid wall thickness was measured between the blood–intima and media–adventitia interfaces by B-mode ultrasonography using software calipers at points of protrusion. Long-axis measures were confirmed by short-axis assessment. The maximum carotid wall thickness for each subject was divided by age in years to yield an annual accretion rate (called carotid intima–media thickness accretion rate [CIMTAR]). The entire study population was then divided by median CIMTAR to investigate the association with baseline variables used in standard risk assessments with the bifurcated groups. Traditional risk factors such as age, diabetes, smoking, hyperlipidemia, and obesity were not associated with greater than median CIMTAR. Only male gender (*P* = 0.02) and systolic blood pressure (*P* = 0.002) in baseline variables were associated with an elevated CIMTAR for the entire population. Among those not taking lipid-lowering therapy at baseline, only systolic blood pressure remained significant (*P* = 0.0002). Correlations between low-density lipoprotein (LDL) cholesterol level and maximum carotid wall thickness/CIMTAR were weak for the entire population (*r* = −0.17/*r* = −0.12, respectively). Measure of maximum carotid wall thickness may select patients earlier for treatment than traditional risk factors. The addition of CIMTAR to risk algorithms may permit a single-point assignation of subsequent vascular risk that is more efficacious than traditional risk factors.

## Introduction

Vascular outcomes such as stroke, acute coronary syndrome, and sudden death are more common with increasing age (American Heart Association, Heart Disease & Stroke Statistics 2007), making age a powerful contributor to risk (http://hp2010.nhlbihin.net/atpiii/calculator.asp). As atherogenesis is a time-dependent process, preclinical measures of vascular changes may identify subjects at highest risk of cardiovascular disease (CVD). Carotid intima–media thickness (CIMT) is a validated surrogate marker of future risk as increasing carotid wall thickness is reflective of the increasing burden of atherosclerosis (Bots et al. 1997; Chambless et al. 1997). Studies of CIMT have generally included subjects aged 45 years and older and are therefore closer to eventual vascular outcomes. In these studies, CIMT was measured over the distal 1 cm of the terminal common carotid artery (CCA) free of plaque. An alternative strategy would be to direct ultrasound measures of wall thickness to identified areas of protrusion. We hypothesized that utilizing such a measure might identify subjects of increased vascular risk at an earlier age perhaps before the onset of risk factors.

## Methods

### Subjects

**Figure 1:**
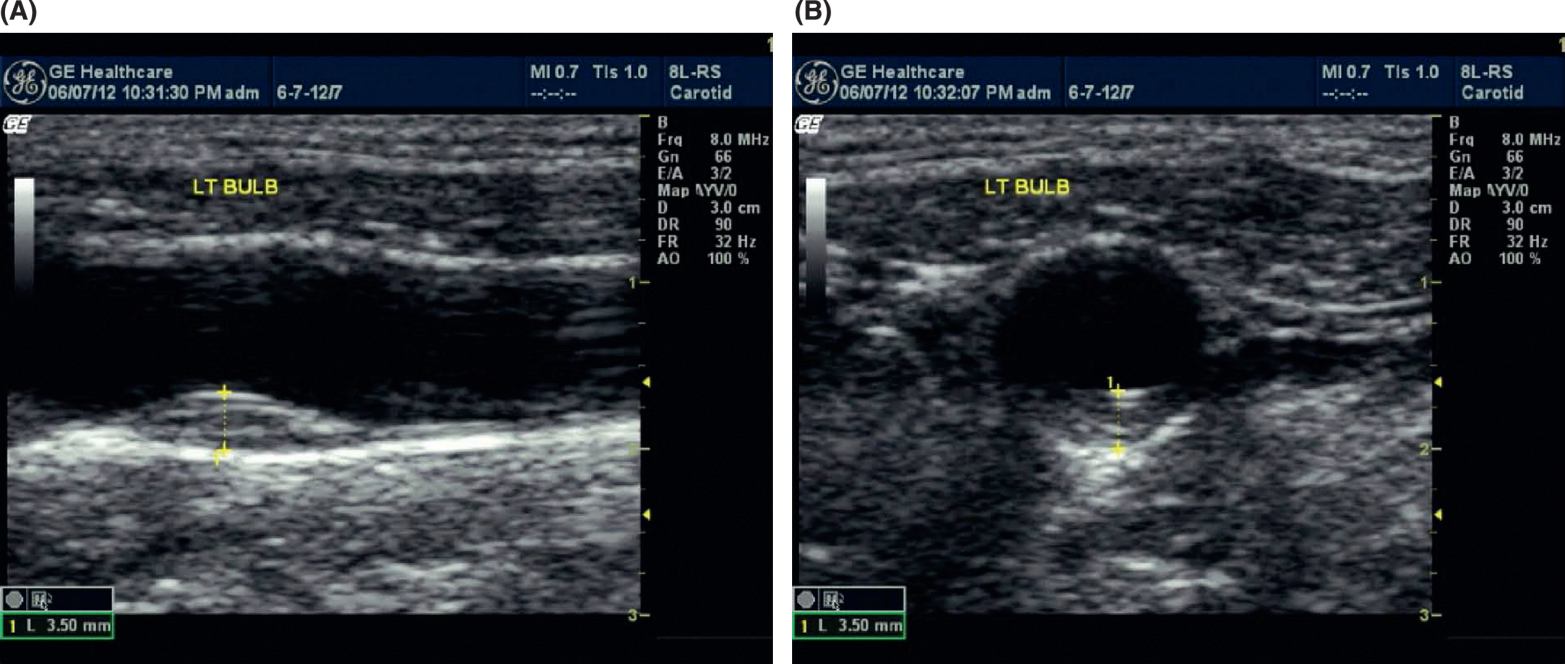
Long-axis B-mode (A) shows software caliper placement between blood–intima and media–adventitia interfaces. The 3.50-mm max wall thickness is confirmed on short-axis view (B).

Subjects without known vascular disease were referred to a vascular medicine clinic for consultation by primary care providers and underwent evaluation of traditional risk factors by history taking, physical examination, laboratory testing, and ultrasound studies. Current and recent past smokers who quit less than 10 years prior were classified as smokers. Between 2007 and 2009, *n* = 393 consecutive subjects were evaluated. The carotid walls were visualized using high-resolution B-mode ultrasonography of the common carotid and internal carotid arteries (CCA and ICA, respectively) bilaterally (GE Logic Book XP, 7.5-mHz linear transducer). Long- and short-axis views were utilized to identify protrusions of the arterial wall into the lumen. Software cursors were manually placed at the blood–endothelial interface and at the media–adventitia junction yielding the measured carotid wall thickness on long-axis views of these protrusions. Short-axis examination was utilized to insure accuracy of each long-axis measurement point (Stein et al. 2008). Cardiac gating was not utilized. The maximum wall thickness was defined as the greatest value from the ensemble of measures (see Fig. 1). A single sonographer certified by the Registry of Diagnostic Medical Sonographers conducted all of the examinations. A single reviewer (ASC) selected the maximum carotid wall thickness after review of all images/measures. An apparent rate of carotid wall thickness increase (CIMT accretion rate [CIMTAR]) was calculated by dividing the maximum value in millimeters by the subject's age in years.

### Statistical analysis

Subjects were classified based on the median CIMTAR for the entire sample. Group comparisons for the entire sample and for the subset not taking lipid-lowering therapy at baseline were made by *t*-tests or chi-square tests. Pearson correlations between maximum wall thickness, CIMTAR, and low-density lipoprotein cholesterol (LDL-C) were also estimated. *P*-values <0.05, two-tailed, were considered statistically significant, with no adjustment for multiple testing.

## Results

**Figure 2:**
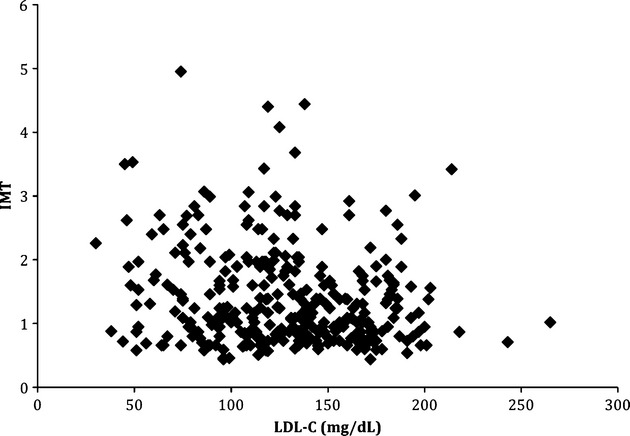
LDL-C versus IMT – all patients (*r* = −0.17, *P* = 0.002). For patients not taking lipid-lowering therapy: *r* = −0.12, *P* = 0.07. LDL-C, low-density lipoprotein cholesterol; IMT, intima–media thickness.

**Figure 3:**
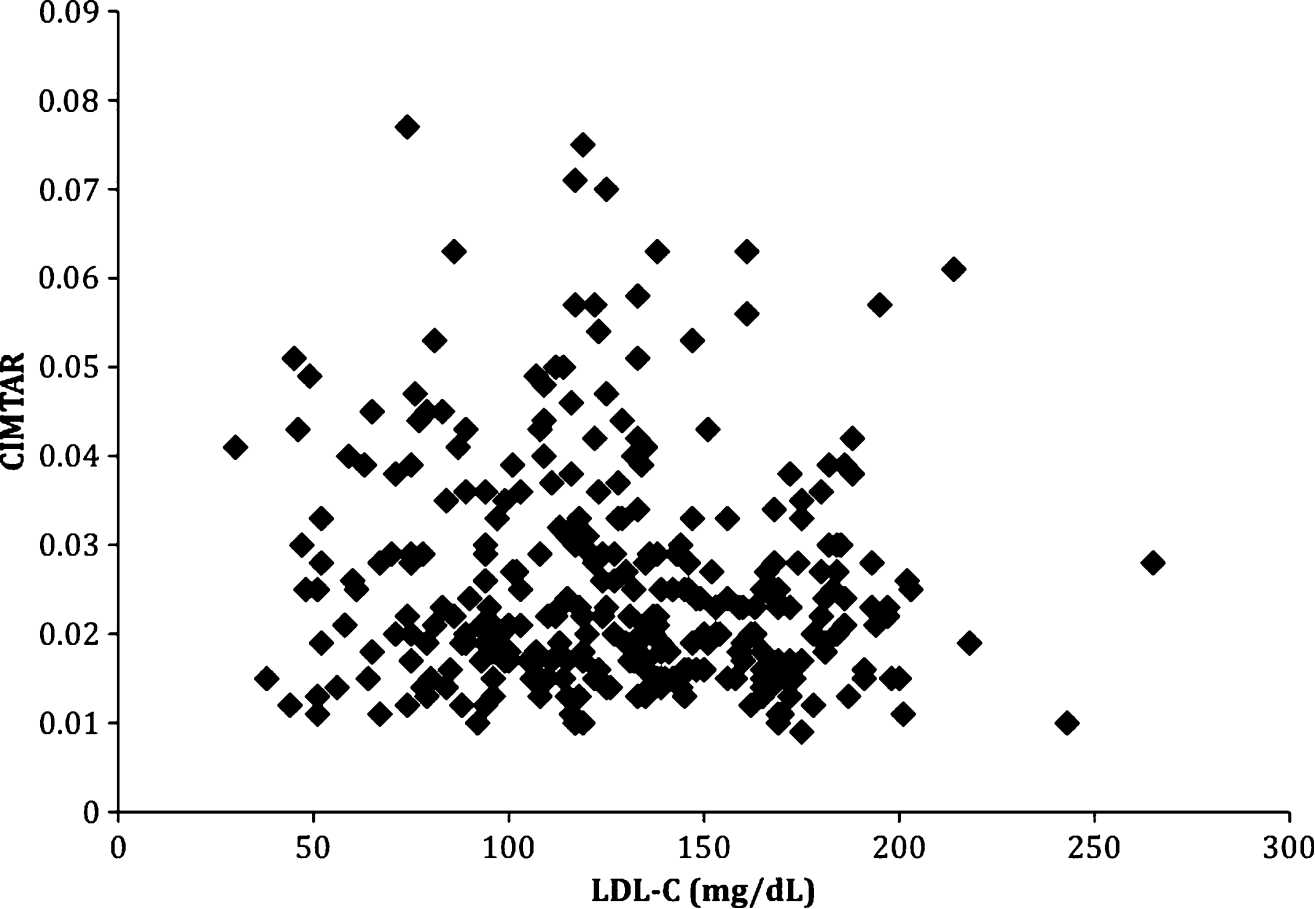
LDL-C versus CIMTAR – all patients (*r* = −0.12, *P* = 0.03). For patients not taking lipid-lowering therapy: *r* = −0.06, *P* = 0.38. LDL-C, low-density lipoprotein cholesterol; CIMTAR, carotid intima–media thickness accretion rate.

The baseline characteristics of the 393 subjects are delineated in Table 1. The duration of known risk factors was not captured at baseline only their presence. There was only a single African-American subject in the data set. Ninety-two (23%) subjects were taking lipid-lowering therapy at baseline. The baseline characteristics of those not taking lipid lowering at baseline are shown in Table 2. Maximum wall thickness and CIMTAR values were similar in the overall group and the statin naïve population (1.38, 1.32 mm; 0.026, 0.025 mm/year, respectively). A comparison of baseline characteristics, maximum wall thickness, and CIMTAR in several recent randomized clinical trials (O'Leary et al. 1999; Crouse et al. 2007; Kastelain et al. 2008) and our results is shown in Table 3. The results of dividing our study population into above and below median CIMTAR values are shown in the second and third columns of Tables 1 and 2. For the overall population, male gender and systolic blood pressure are the only baseline characteristics that are statistically associated with elevated CIMTAR among traditional risk factors (*P* = 0.02, 0.002, respectively). Among those not taking lipid-lowering therapy, only systolic blood pressure remains significant (*P* = 0.0002). There was a statistically significant association between maximum wall thickness and baseline LDL for the entire population (*P* = 0.002), but the association is a negative one with a weak correlation (*r* = −0.17; Fig. 2). The association was not significant for the subjects not on lipid-lowering therapy (*P* = 0.07, *r* = −0.12). Scatter plots of CIMTAR and baseline LDL for the entire population were only weakly associated (*P* = 0.03, *r* = −0.12; Fig. 3). Again, the association was not significant for subjects not on lipid-lowering therapy (*P* = 0.38, *r* = −0.06).

**Table 1:** Baseline patient characteristics – all patients

	All patients (*n* = 393)	≤Median CIMTAR (*n* = 202)	>Median CIMTAR (*n* = 191)	*P*-value
Age (years)	54 ± 10	53 ± 11	55 ± 10	0.10
Male gender	48	42	54	0.02
Systolic blood pressure (mmHg)	127 ± 15	125 ± 13	130 ± 16	0.002
Diastolic blood pressure (mmHg)	81 ± 11	80 ± 11	82 ± 10	0.16
Body mass index (kg/m^2^)	29 ± 12	29 ± 6	30 ± 15	0.32
LDL-C (mg/dL)	128 ± 41	130 ± 40	127 ± 42	0.43
HDL-C (mg/dL)	56 ± 42	55 ± 17	57 ± 57	0.62
Triglyceride (mg/dL)	140 ± 80	140 ± 75	139 ± 85	0.90
FBG	95 ± 23	93 ± 14	97 ± 29	0.15
Smoking	14	12	16	0.26
Diabetes	6	4	8	0.11
CIMT	1.38 ± 0.77	0.85 ± 0.23	1.93 ± 0.76	<0.0001
CIMTAR	0.026 ± 0.013	0.016 ± 0.003	0.035 ± 0.012	<0.0001

Values in the table are mean ± SD or %. *P*-values are from *t*-tests or chi-square tests of CITMAR median category comparisons. Note: Median CIMTAR = 0.0220. CIMTAR, carotid intima–media thickness accretion rate; LDL-C, low-density lipoprotein cholesterol; HDL-C, high-density lipoprotein cholesterol; FBG, fasting blood glucose; CIMT, carotid intima–media thickness.

**Table 2:** Baseline patient characteristics – patients not taking lipid-lowering therapy

	All patients (*n* = 301)	≤Median CIMTAR (*n* = 154)	>Median CIMTAR (*n* = 147)	*P*-value
Age (years)	53 ± 11	53 ± 10	53 ± 11	0.98
Male gender	49	46	52	0.24
Systolic blood pressure (mmHg)	127 ± 15	124 ± 13	130 ± 17	0.0002
Diastolic blood pressure (mmHg)	81 ± 11	79 ± 11	82 ± 10	0.04
Body mass index (kg/m^2^)	30 ± 13	29 ± 6	30 ± 17	0.35
LDL-C (mg/dL)	128 ± 42	129 ± 42	126 ± 42	0.67
HDL-C (mg/dL)	53 ± 16	53 ± 15	53 ± 16	0.92
Triglyceride (mg/dL)	140 ± 81	141 ± 76	140 ± 85	0.91
FBG	96 ± 25	93 ± 13	98 ± 32	0.16
Smoking	13	14	12	0.25
Diabetes	7	5	9	0.80
CIMT	1.32 ± 0.74	0.82 ± 0.21	1.81 ± 0.76	<0.0001
CIMTAR	0.025 ± 0.013	0.016 ± 0.003	0.034 ± 0.012	<0.0001

Values in the table are mean ± SD or %. *P*-values are from *t*-tests or chi-square tests of CITMAR median category comparisons. Note: Median CIMTAR = 0.0215. CIMTAR, carotid intima–media thickness accretion rate; LDL-C, low-density lipoprotein cholesterol; HDL-C, high-density lipoprotein cholesterol; FBG, fasting blood glucose; CIMT, carotid intima–media thickness.

**Table 3:** Maximum carotid wall thickness in other randomized trials

	CHS (O'Leary et al. 1999)	METEOR (Crouse et al. 2007)	ENHANCE (Kastelain et al. 2008)
Age (mean, years)	72.5	57	45
LDL (mean, mg/dL)	130	155	193
CIMT CCA (mm)	1.03	1.02	0.68
CIMTAR CCA (mm/year)	0.014	0.018	0.015
CIMT ICA (mm)	1.37	1.06	0.62
CIMTAR ICA (mm/year)	0.019	0.019	0.014

Study population in CHS was adults >65 years without vascular disease. Mean of maximum CIMT was taken from their Table 1 (near plus far wall on both sides). METEOR enrolled low-risk (<10% 10-year risk of vascular disease) subjects. The mean values in the enhance study were taken from their Table 3. They had a single value for the mean of maximum CIMT which yields a CIMTAR of 0.018 mm/year. LDL, low-density lipoprotein; CCA, common carotid artery; ICA, internal carotid artery; CIMT, carotid intima–media thickness; CIMTAR, carotid intima–media thickness accretion rate.

## Discussion

One advantage of risk attribution by a single measure at any point in time such as CIMTAR would be to select subjects earlier in life without having to wait for the time-based increase in wall thickness to pass a threshold beyond normal. Such a single measure might also select subjects at risk prior to the development of risk factors. The similar values of CIMTAR from trials of differing patient populations (older, low risk, or young with familial hypercholesterolemia) suggest a linear accretion of wall thickness independent of baseline LDL-C levels and support the validity of the concept of a snapshot determination of risk. The larger mean CIMTAR in our population may be a result of the selection of the maximum CIMT for each subject and increased baseline risk in the referral population. A CIMTAR of ≤0.016 mm/year may prove to be a useful cutpoint for populations such as ours.

At every level of baseline LDL-C, half of the population was variably distributed above the median CIMTAR, while the other half was densely grouped below. Between these two groups, traditional risk factors did not account for their separation, and baseline LDL was not associated with elevated maximum wall thickness or CIMTAR. The increase in carotid wall thickness was not determined by the concentration gradient of LDL between serum and the subendothelial space. In previous studies (Table 3), similar CIMTAR values were noted despite varying mean LDL levels suggesting that factor(s) other than the LDL gradient determine maximum wall thickness. Potential contributors to an excess wall thickness might be trafficking of lipoproteins in the arterial wall. However, in our patient population, high-density lipoprotein (HDL) and triglyceride (TG) levels were also comparable between the two groups. Another possibility may be that variability in vascular endothelium barrier properties contributed to the excess in wall thickness and apparent accretion rate.

The single baseline variable associated with an elevated CIMTAR for both the overall population and those without lipid-lowering therapy at baseline one was systolic blood pressure. In meta-analyses of hypertensive trials, elevations in systolic blood pressure were associated with an increase in risk of vascular outcomes with a 40% increment for every 10-mmHg increase (Chobanian et al. 2009). There are multiple mechanisms by which hypertension may increase maximum wall thickness: increased lipid entry into the subendothelial layer, loss of smooth muscle architecture with hyperplasia/dedifferentiation/lipid ingestion, and increases in lipid oxidation, inflammation, and peptidergic signaling among others (Mulvany and Aalkjaer 1990; Ross 1999). Alternatively, the increase in systolic blood pressure in the above median CIMTAR group could be from cytoarchitectural change in the distal arterioles.

Limitations of our study include the operator dependence of ultrasound measures. Although automated means of maximum wall thickness measure may help reduce operator error, we used short-axis examination to confirm the longitudinal measurements. The addition of morphologic measures of the content of the carotid wall such as grayscale median (GSM) might enhance the accuracy of risk stratification (Wohlin et al. 2009; Graebe et al. 2010). Serial follow-up of a primary prevention population would be necessary to establish the clinical utility of CIMTAR. However, recent results from the Framingham Offspring Study support the use of maximum wall thickness to enhance risk stratification (Polak et al. 2011).

Earlier identification of vascular risk by a single imaging measure such as CIMTAR may enable earlier treatment and expanded benefit from a longer duration of care. Enhanced communication of such risk may increase adherence to risk reduction programs, which is critical for long-term or lifetime treatment strategies. There is abundant need for more efficient treatments of larger patient populations to reduce vascular outcomes such as acute coronary syndrome, stroke, and sudden death.
